# The effects of early-life growth hormone intervention on tissue specific histone H3 modifications in long-lived Ames dwarf mice

**DOI:** 10.18632/aging.202451

**Published:** 2020-12-28

**Authors:** Fang Zhang, Mert Icyuz, Andrzej Bartke, Liou Y. Sun

**Affiliations:** 1 Department of Biology, University of Alabama at Birmingham, Birmingham, AL 35254, USA; 2 Department of Internal Medicine, Southern Illinois University School of Medicine, Springfield, IL 62702, USA

**Keywords:** Ames dwarf mice, growth hormone, DNA methylation, histone H3 methylation, histone H3 acetylation

## Abstract

Histone modifications, specifically in the lysine residues of histone H3, have been implicated in lifespan regulation in several model organisms. Our previous studies showed that growth hormone (GH) treatment during early life can dramatically influence lifespan in long-lived Ames dwarf mice. However, the effects of this hormonal intervention on epigenetic modifications have never been examined. In this study, we sought to compare tissue-specific histone H3 lysine methylation and acetylation markers in Ames dwarf and wild type (WT) mice and to determine how these markers are affected by early-life GH intervention. Ames dwarf mice exhibited suppressed H3K4me in both hepatic and brain tissues, while showing elevated H3K27me in the brain. Early-life GH intervention significantly altered the histone H3 markers in those tissues. Furthermore, early GH intervention increased expression of histone H3 acetylation at multiple lysine residues in a tissue-specific manner. This included changes in H3K14ac and H3K18ac in the liver and brain, H3K18ac in visceral adipose tissue and H3K9ac, H3K14ac and H3K27ac in subcutaneous adipose tissue. This study serves as an initial, but important step in elucidating the epigenetic mechanisms by which hormonal signals during early life can influence aging and longevity in mammals.

## INTRODUCTION

Actions of growth hormone (GH) during the rapid period of peri-pubertal growth shapes the trajectory of aging. This novel action of GH was discovered by treating GH-deficient Ames dwarf mice with GH for a relatively short period (six weeks) early in their postnatal life. This hormonal intervention led to persistent (likely permanent) changes in phenotypic characteristics associated with healthy aging including insulin, glucose level, adiponectin levels and expression of genes related to xenobiotic metabolism and stress responses [1]. Importantly, treating juvenile Ames dwarf mice with GH partially shortened the remarkably extended longevity of these animals [1]. Subsequent studies provided evidence that early life GH intervention in Ames dwarfs mice rescues (normalizes) hypothalamic gliosis [2], NLRP3 inflammasome activity [3] and, hepatic production of hydrogen sulfide [4]. We now seek to identify the mechanisms of these presumably epigenetic effects. As the first step in this direction, we have examined histone H3 modifications in Ames dwarfs treated with GH injections for six weeks starting at two weeks of age along with dwarfs treated with vehicle and wild type controls.

Aging and age-related diseases can be regulated epigenetically by specific alterations in DNA methylation and histone H3 modifications pattern. DNA methyltransferase 1 (DNMT1), DNA methyltransferase 3α (DNMT3α) and DNA methyltransferase 3β (DNMT3β) gradually decreases with aging in mammals [5]. Decrease in H3K4 methyltransferase (H3K4me) and H3K27 tri-methyltransferase (H3K4me3) are linked to enhanced longevity in *C. elegans* and fruit flies [6–8]. Acetylations of lysine 18 (H3K18ac) and 56 (H3K56Ac) in histone H3 act as negative markers of aging in fruit flies [9] and yeast [10], respectively. In rodents, aging significantly decreases hepatic expression of acetylation at lysine 9 of histone H3 (H3K9ac) [11] and leads to a dynamic feature at lysine 27 of histone H3 (H3K27ac) in the liver and brain [12]. H3K14ac level was shown to regulate aging-related synaptic plasticity gene expression in aging mice brain [13]. In human cortex, aging-mediated changes in H3K9ac and H3K27ac are associated with Alzheimer’s disease (AD) [14, 15].

In Ames dwarf mice (*Prop1^df/df^*), a well-established, long-lived and GH deficient mouse model, the hepatic level of DNMTs was higher in the young age and lower in old age compared with age-matched controls [16]. Intriguingly, acute administration of porcine GH markedly increased DNMT1 and decreased DNMT3α levels in Ames dwarf mice [16]. In addition, hypo- and hyper-methylations in the liver were associated with aging in Ames dwarf mice [17]. Our previous data showed that GH intervention during early development shortened longevity in the long-lived Ames dwarf mice [1]. However, its effect on epigenetic modifications has never been reported. In this study, we sought to examine tissue-specific histone H3 lysine methylation and acetylation markers in Ames dwarf mice upon early-life GH intervention. This study is intended to contribute an initial, but important step in the understanding of the epigenetic mechanisms of early-life hormonal regulation of aging and longevity in mammals.

## RESULTS

### GH early-life intervention regulates histone H3 methylation in the liver

**Figure 1:**
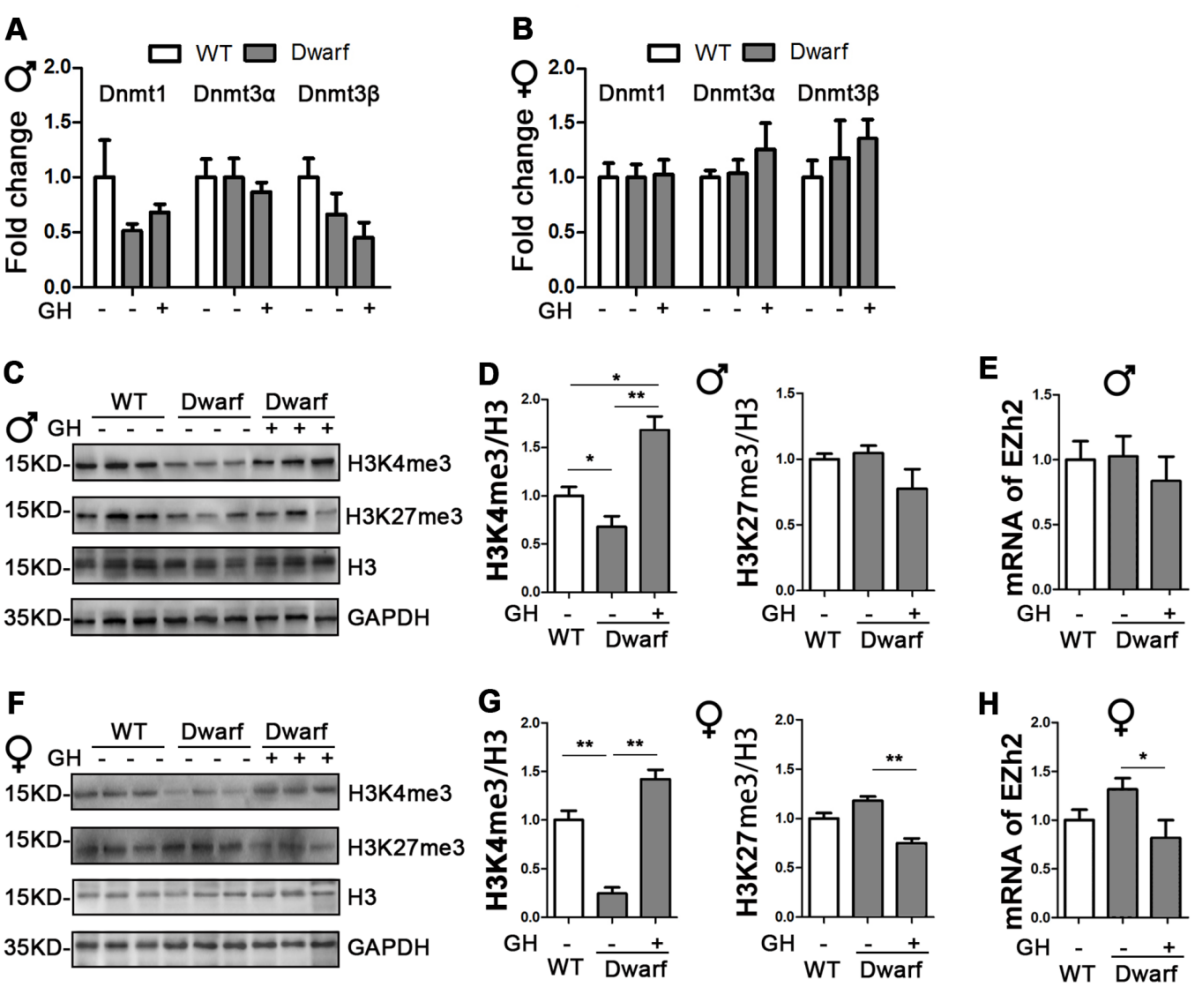
**Impact of early life GH intervention on hepatic expressions of DNMTs and H3 methylation on lysine 4 and 9 in Ames dwarf mice.** (**A**, **B**) Hepatic DNMTs mRNA expression is shown for 20-month-old males (**A**) and females (**B**). (**C**, **F**) Representative western blots in males (**C**) and females (**F**). (**D**, **G**) Quantification of hepatic H3K4me3 and H3K27me3 in Ames dwarf males (**D**) and females (**G**) mice upon early-life GH intervention. (**E**, **H**) mRNA level of EZH2 in males (**E**) and females (**H**) of Ames dwarf mice. mRNA analysis data (means ± sem) (**A**, **B**, **E**, **H**) are normalized to GAPDH and expressed as fold change compared with wild type (WT) control (defined as 1.0). Protein quantification data (means ± SEM) (**D**, **G**) are normalized to histone H3 and expressed as fold change compared with WT control (defined as 1.0), n=6 mice for each group. Data are means ± SEM. * *p* < 0.05, ** *p* < 0.01 by one-way ANOVA.

Our previous data indicated that early-life GH intervention significantly shortened longevity and impaired health related outcomes in Ames dwarf mice [1]. Long-lived Ames mice have altered DNMTs-mediated DNA methylation pattern during aging [16, 17]. To test whether exposure to GH at early life affects DNA methylation enzymes, real-time quantitative PCR analysis of isolated RNA was performed to examine hepatic expression of DNMTs from Ames dwarf mice injected with GH or saline starting at the age of 2 weeks and continuing for 6 weeks. Mice were euthanized for tissue collection after the GH intervention was completed and the animals were 20 months old. We found similar expression levels of DNMTs between Ames dwarf mice and controls (Figure 1A, 1B), suggesting that early-life GH intervention did not change mRNA level of DNMTs in the liver of Ames dwarf mice.

Histone H3 methylation on lysine residues plays an important role in aging and aging-related diseases [8]. Therefore, we examined aging-related histone H3 tri-methylation at lysine 4 and 27 residues in the liver. We found that protein level of H3K4me3 were significantly decreased in Ames dwarf mice compared with controls in both sexes (Figure 1C, 1D, 1F, 1G and Supplementary Figure 1). However, early-life GH intervention enhanced activation of H3K4me3 in both female and male Ames dwarf mice. This was especially pronounced in males in which that H3K4me3 was 1.6-fold increase in Ames dwarf mice compared with controls (Figure 1C, 1D, 1F, 1G and Supplementary Figure 1). Similar expression of H3K27me3 was observed in both Ames dwarf and control mice (Figure 1C, 1D, 1F, 1G and Supplementary Figure 1). Furthermore, early-life GH treatment led to a significant decrease in H3K27me3 level in females, while no difference was found in males (Figure 1F, 1G and Supplementary Figure 1). The tri-methylation of H3K27 is mediated by EZH2 to suppress gene expression [18]. We found that the mRNA level of EZH2 was decreased 40 % in female Ames dwarf mice upon early-life GH intervention (Figure 1E–1H).

### GH early-life intervention changes histone H3 methylation in the brain

**Figure 2:**
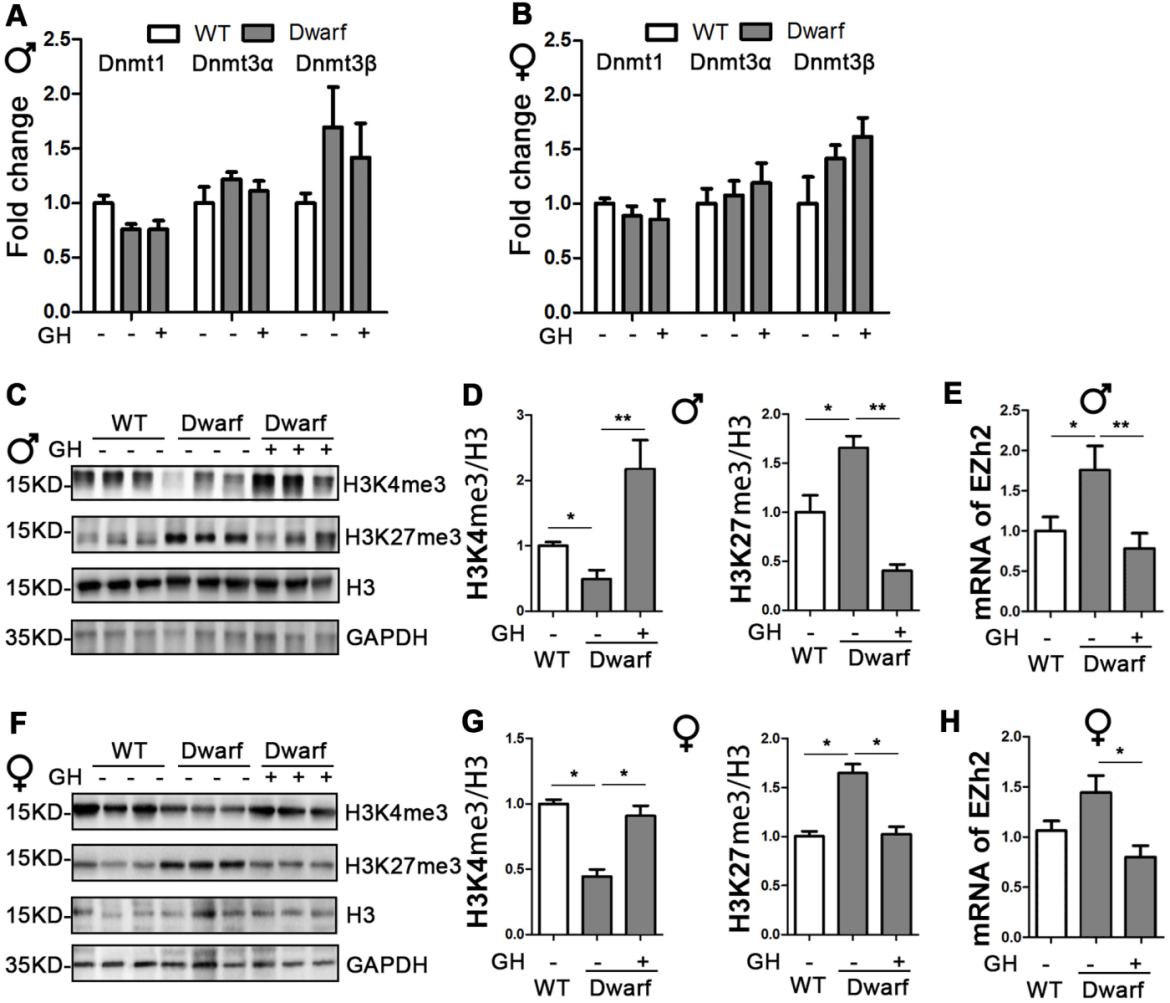
**Early-life GH intervention effects brain expression of DNMTs and H3 methylation on lysine 4 and 27 in Ames dwarf mice.** (**A**, **B**) Brain DNMTs expression is shown for 20-month-old males (**A**) and females (**B**). (**C**, **F**) Representative western blots in males (**C**) and females (**F**). (**D**, **G**) Quantification of brain H3K4me3 and H3K27me3 in Ames dwarf males (**D**) and females (**G**) mice upon early-life GH intervention. (**E**, **H**) mRNA level of EZH2 in males (**E**) and females (**H**) of Ames dwarf mice. mRNA analysis data (means ± sem) (**A**, **B**, **E**–**H**) are normalized to GAPDH and expressed as fold change compared with WT control (defined as 1.0). Protein quantification data (means ± SEM) (**D**, **G**) are normalized to histone H3 and expressed as fold change compared with WT control (defined as 1.0). n=6 mice for each group. Data are means ± SEM. * *p* < 0.05, ** *p* < 0.01 by one-way ANOVA.

DNA methylation and histone H3 methylation changes are associated with aging and aging-related neurodegenerative diseases, including AD [19]. Therefore, we examined the mRNA level of DNMTs in the brain. We found similar expressions of DNMTs between Ames dwarf mice and controls in both sexes (Figure 2A, 2B). We next found the markedly decreased H3K4me3 and increased H3K27me3 in the brain of Ames dwarf mice compared with controls (Figure 2C, 2D, 2F, 2G and Supplementary Figure 2). The expression of H3K4me3 in the brain was increased 4.6-fold in male and 2-fold in female Ames dwarf mice upon early-life GH intervention (Figure 2C, 2D, 2F, 2G and Supplementary Figure 2). Moreover, the activation of H3K27me3 was reduced by early-life GH treatment in Ames dwarf mice (5.3-fold in males versus 1.7-fold in females, Figure 2C, 2D, 2F, 2G). The brain mRNA level of EZH2 was increased in male Ames dwarf mice compared with controls, however, EZH2 was reduced 2-fold by GH early-life intervention in Ames dwarf mice (Figure 2E, 2H).

### Hepatic histone H3 acetylation changes in Ames dwarf mice upon GH intervention

**Figure 3:**
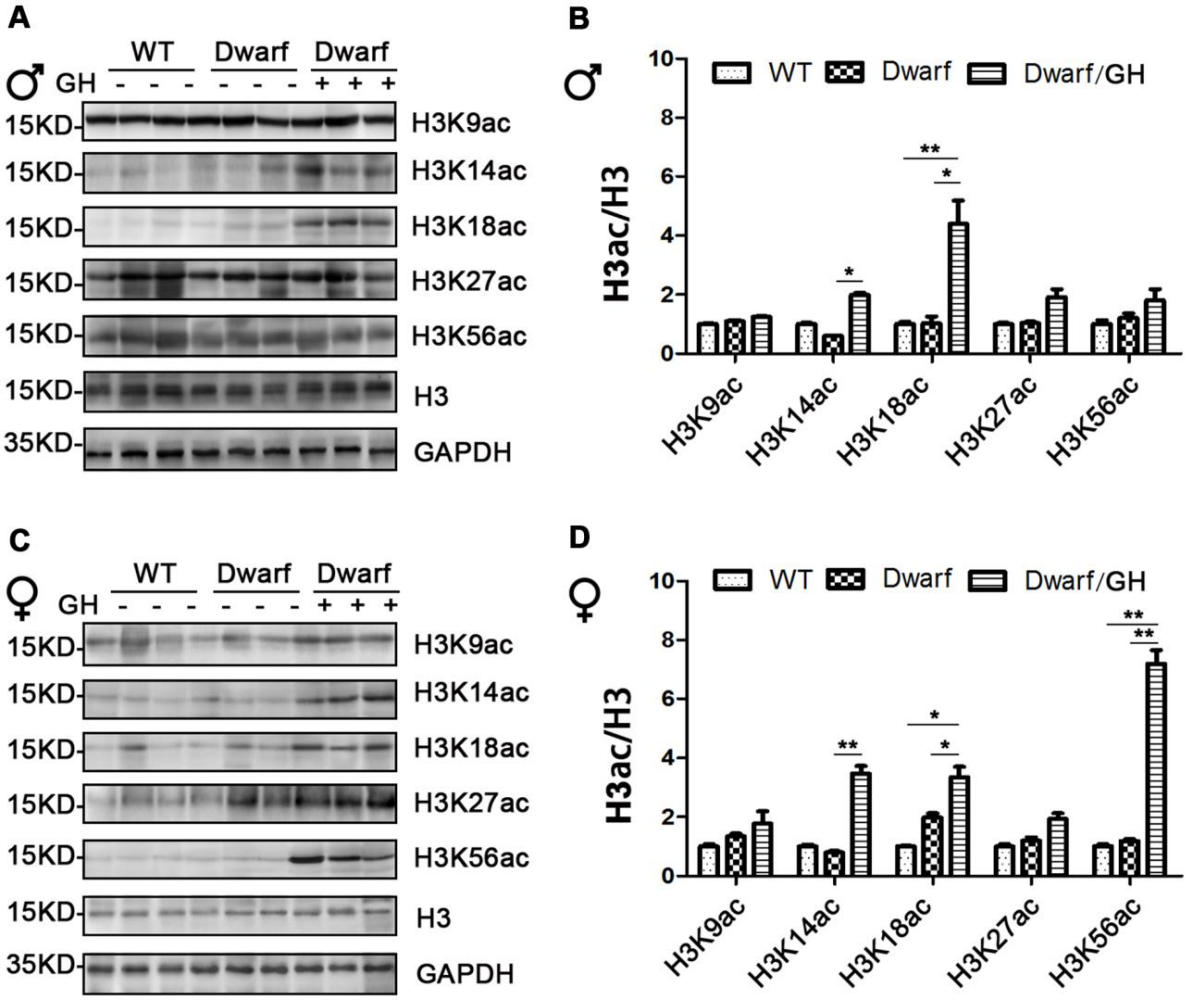
**Hepatic histone H3 acetylation changes in Ames dwarf mice upon early-life GH intervention.** (**A, C**) Representative western blots in Ames dwarf males (**A**) and females (**C**) mice upon early-life GH intervention. (**B**, **D**) Quantification of acetylation of histone H3 in males (**B**) and females (**D**). Protein quantification data (means ± SEM) are normalized to histone H3 and expressed as fold change compared with WT control (defined as 1.0), n=6 mice for each group. Data are means ± SEM. * *p* < 0.05, ** *p* < 0.01 by one-way ANOVA.

Genome-wide histone H3 acetylation at multiple lysine residues usually functions as positive regulator during aging [20]. To further understand the relationship between histone acetylation and gene regulation in aging, we investigated the site-specific acetylation of histone H3 in the liver of Ames dwarf mice. The expression of H3K9ac, H3K14ac, H3K18ac, H3K27ac and H3K56ac in the liver was similar between Ames dwarf mice and controls of both sexes (Figure 3 and Supplementary Figure 3). However, early life GH intervention significantly increased activation of H3K14ac and H3K18ac in males (4.2-fold in H3K14ac and H3K18ac, respectively) and females (4-fold in H3K14ac and 1.7-fold in H3K18ac, Figure 3 and Supplementary Figure 3). Additionally, expression of H3K56ac was significantly increased (7-fold) by GH intervention in early age in male Ames dwarf mice compared with controls (Figure 3C, 3D and Supplementary Figure 3).

### Prolife of histone H3 acetylation in the brain of Ames dwarf mice upon GH early-life intervention

**Figure 4:**
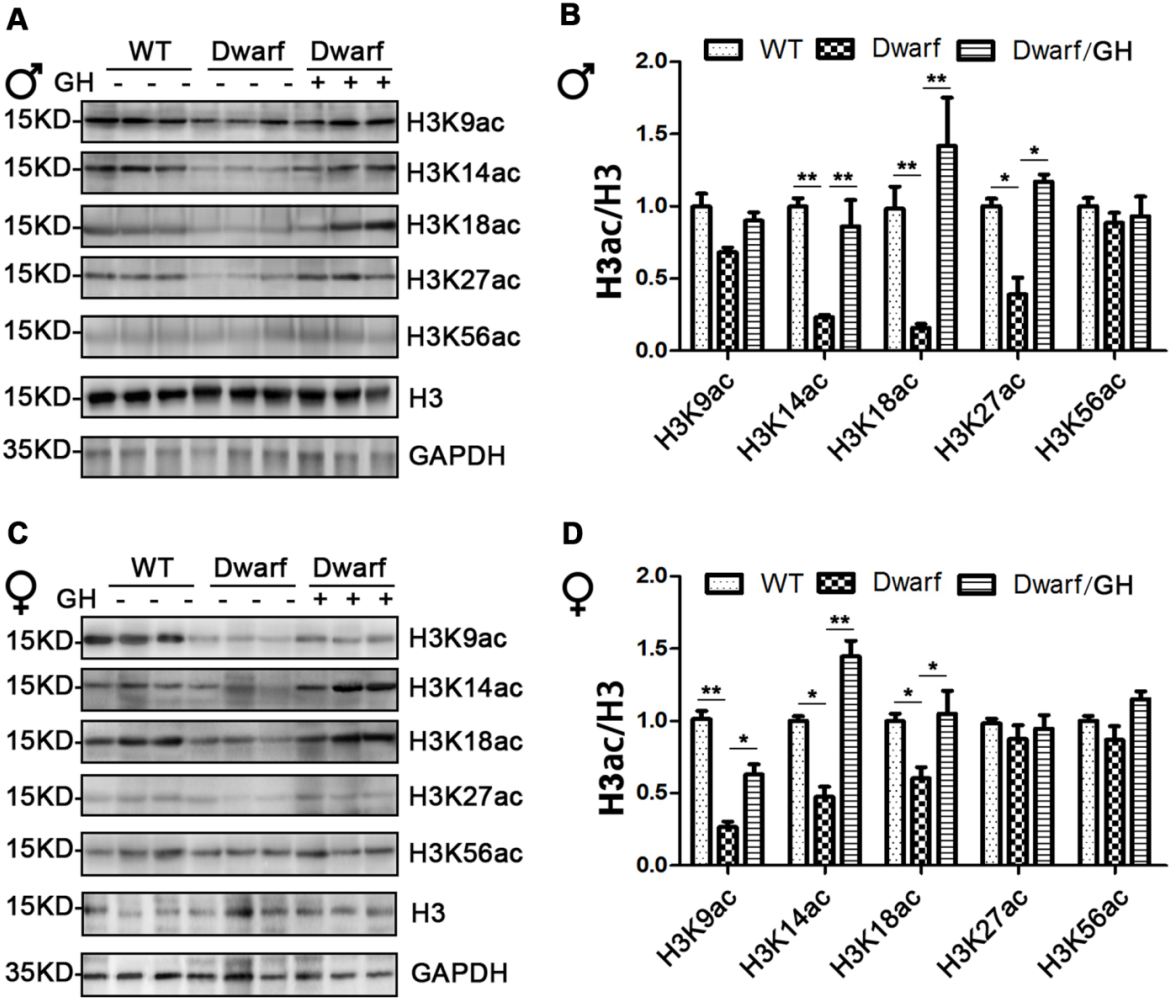
**Brain histone H3 acetylation altered in Ames dwarf mice upon early-life GH intervention.** (**A**, **C**) Representative western blots in males (**A**) and females (**C**) mice upon early-life GH intervention. (**B**–**D**) Quantification of acetylation of histone H3 in males (**B**) and females (**D**). Protein quantification data (means ± SEM) are normalized to histone H3 and expressed as fold change compared with WT control (defined as 1.0), n=6 mice for each group. Data are means ± SEM. * *p* < 0.05, ** *p* < 0.01 by one-way ANOVA.

We next focused on histone H3 acetylation in the brain regions of Ames dwarf mice because the brain is known to display age-related functional decline that can lead to neurodegenerative diseases. Expression of H3K14ac and H3K18ac in cerebral cortex was markedly decreased in Ames dwarf mice of both sexes (3-fold in H3K14ac and 5-fold in H3K18ac in males, 2-fold in H3K14ac and 1.3-fold in H3K18ac in females), H3K27ac was reduced 2-fold in males and H3K9ac was reduced 3-fold in females of Ames dwarf (Figure 4). However, early-life GH intervention activated the acetylation of H3K14, H3K18 and H3K27 in males and H3K9, H3K14 and H3K18 in females, and this increased acetylation was similar to the values measured in the control cerebral cortex (Figure 4 and Supplementary Figure 4).

### Early-life GH intervention changes acetylation of histone H3 in white adipose tissues in Ames dwarf mice

**Figure 5:**
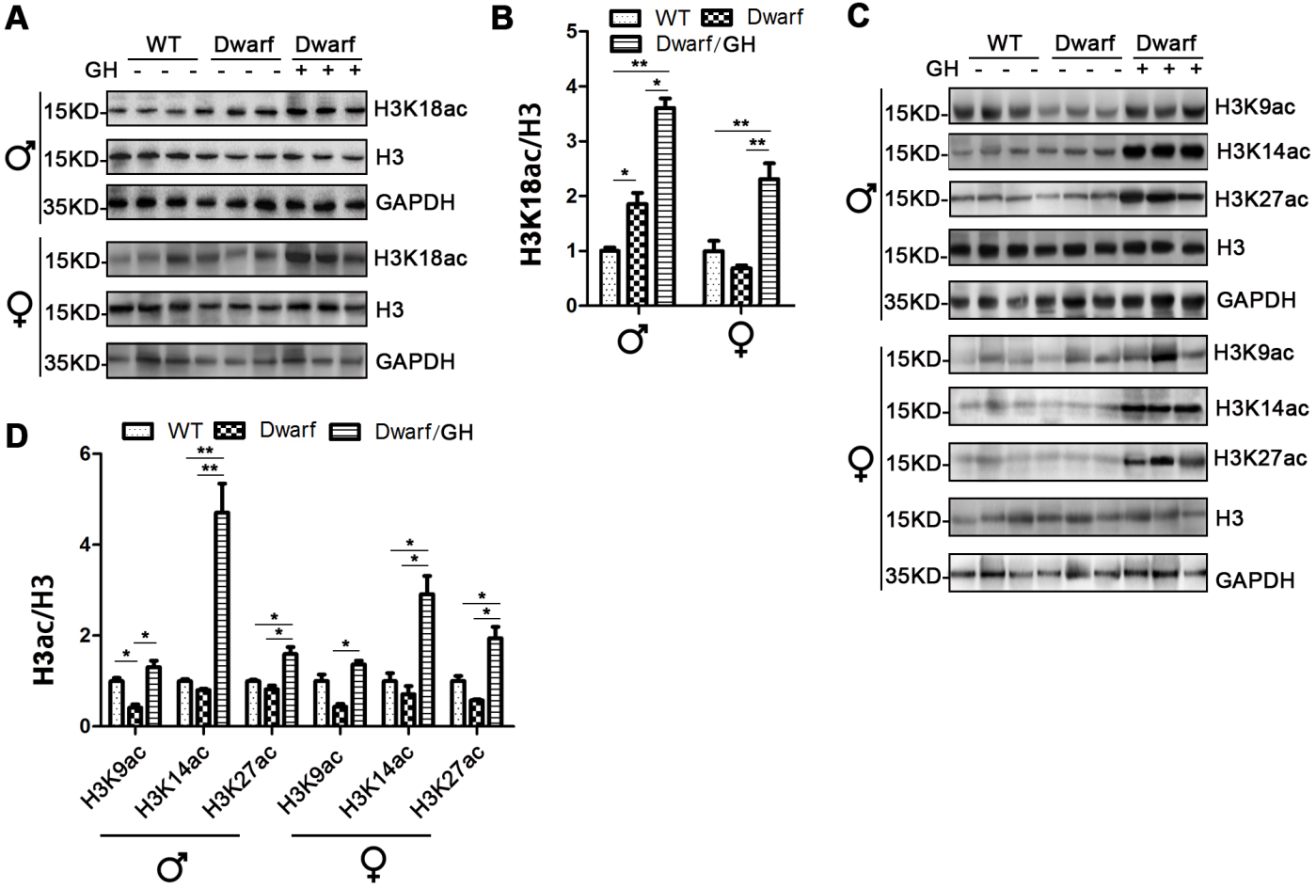
**Histone H3 acetylation changes in white adipose tissues of Ames dwarf mice upon GH intervention at early age.** (**A**, **C**) Representative western blots in visceral adipose tissue (**A**) and subcutaneous adipose tissue (**C**) in mice upon early-life GH intervention. (**B**, **D**) Quantification of acetylation of histone H3 in visceral adipose tissue (**B**) and subcutaneous adipose tissue (**D**). Protein quantification data (means ± SEM) are normalized to histone H3 and expressed as fold change compared with WT control (defined as 1.0), n=6 mice for each group. Data are means ± SEM. * *p* < 0.05, ** *p* < 0.01 by one-way ANOVA.

Attenuated or slow aging-related increase in adipose tissues of Ames dwarf mice implied that dwarfs are biologically younger compared with chronological age-matched controls [21]. Because white adipose tissues account for the majority of fat mass, it is critical to understand aging-related profile of histone H3 acetylation in this tissue in healthy aging Ames dwarf mice. We profiled H3 acetylation at various lysine residues in visceral adipose tissue of Ames dwarf mice and found that H3K18ac was elevated 2-fold in male Ames dwarfs compared with controls (Figure 5A, 5B and Supplementary Figure 5). GH intervention at early age further increased the amount of H3K18ac with a 3.7-fold increase in males and 2.5-fold increase in females, and increased H3K18ac to values significantly higher than controls (Figure 5A, 5B and Supplementary Figure 5).

The data of H3 acetylation in subcutaneous adipose tissue showed that expression of H3K9ac was markedly decreased in male Ames dwarf mice compared with controls (Figure 5C, 5D and Supplementary Figure 6). However, the expression of H3K9ac, H3K14ac and H3K27ac was induced in both sexes of Ames dwarf mice upon GH intervention at early age (Figure 5C, 5D and Supplementary Figure 6). Early life GH intervention-induced activation of H3K14ac and H3K27ac was significantly higher in Ames dwarf mice (average 4-fold in H3K14ac and 2-fold in H3K27ac) than in the controls (Figure 5C, 5D and Supplementary Figure 6).

## DISCUSSION

### No influence of early-life GH intervention in expression of DNMTs in Ames dwarf mice

Our data indicated that activation of DNMTs was similar in Ames dwarfs and WT control mice at 20 months of age. These findings are consistent with those of Vanessa et al. showing a much higher hepatic mRNA expression of DNMT1 and DNMT3α in Ames dwarfs than in WT while the increase of DNMT1 was not observed in 12- and 24-month-old mice and hepatic DNMT3β levels were not different between genotypes at 3, 12 and 24 months of age [16]. We have previously shown that early-life GH intervention shortened lifespan in Ames dwarf mice [1]. Therefore, we tested whether expression of DNMTs may be influenced by early-life GH mediated events. Unexpectedly, mRNA level of DNMTs was not significantly altered in Ames dwarf mice by this early-life GH treatment, suggesting that the global level of DNA methylation is highly similar between vehicle and early-life GH treated Ames dwarf mice. However, Adams et al. reported that rapamycin and caloric restriction influence hepatic hypermethylation and hypomethylation in differentially methylated regions in female UM-HET3 mice even though the global DNA methylation is similar in dairy and drug intervention [17].

DNA methylation in the brain is associated with aging-related neurodegenerative diseases [22], in particular, DNMT3α is essential for the formation of memory and synaptic and neuronal plasticity [23]. However, in our study, neither genotype nor early life GH treatment influenced expression of DNMTs in the brain. Since, global DNA methylation is affected by multiple factors, including DNMTs, ten-eleven translocation methylcytosine dioxygenases and methyl-binding protein, it would be interesting to further examine brain DNA methylation in Ames dwarf mice using different markers and methods.

### Early-life GH intervention regulates changes of histone H3 methylation during aging

Histone methylation of lysine residues is among most prominent histone modifications that affect longevity [24]. H3K4me is a negative regulator of longevity in *C. elegans* and aging of stem cells in mice [6, 25]. Here, we found that genetic predisposition to longevity was associated with down-regulation of hepatic H3K4me3 levels. Intriguingly, early-life GH exposure prevented the age-related decline in H3K4me3 in both male and female Ames dwarf mice. These data further support the notion that methylation pattern of H3K4 might have negative impacts on lifespan.

EZH2 acts as histone methyltransferase to regulate hepatic H3K27 methylation pattern in embryonic stem cells, mice, and humans during aging [18, 26]. In our study, we did not find significant differences in hepatic expression of EZH2 or H3K27me3 between Ames dwarf mice and controls in either sex. However, early-life GH intervention decreased EZH2 and H3K27me3 in female mice, suggesting that EZH2 regulates H3K27ac under GH intervention in Ames dwarf mice. Aging is associated with expression of H3K27me in *Drosophila* [8]. Our data showed similar hepatic expression of H3K27me3 in Ames dwarf mice and controls. These data indicate that aging regulates methylation of H3K27 in species-specific manner. During aging, H3K4me is reduced while H3K27me is increased in mouse brain [19]. Anti-aging interventions, such as dietary restriction and rapamycin, prevented age-related alterations of H3K4me and H3K27me in the mouse brain [19]. These data are consistent with our finding that a reverse pattern of H3K4me3/H3K27me3 in the brain of long-lived dwarf mice. Intriguingly, early-life GH treatment normalized these changes. The possible explanation is that methylation of histone H3 on lysine 4 and 27 was affected by this intervention. In addition, we found that the expression of EZH2 was elevated in dwarf mice, and reduced by early-life GH treatment. These data suggest that EZH2 is part of the protein machinery that shapes the aging-related histone H3 methylation level and acts as an important factor in the regulation of expression of genes during aging.

### Early-life GH intervention influences global gene expression via altered age-related histone H3 acetylation

In addition to methylation on histone H3, acetylation on histone 3 positively regulates gene expression in aging [20]. Hepatic H3K9ac was significantly reduced while H3K14ac was not altered in aging rats [11]. Acetylation of H3K18 declines in fruit flies during aging [9]. Our data revealed a similar pattern of differences, between Ames dwarfs and controls in terms of hepatic histone H3 acetylation (multiple lysine residues). This might imply that loss of GH signaling affects the histone H3 acetylation in the liver.

Brain H3K18ac was induced in mice during aging [19]. In the cortex, increased H3K9ac and H3K27ac are associated with AD [14, 15]. Our data showed that H3K14ac, H3K18ac and H3K27ac in males and H3K9ac, H3K14ac and H3K18ac in females were decreased in the brain of Ames dwarf mice. This suggests that histone H3 acetylation might play a more important role in the brain than in the liver. Furthermore, we found that early-life GH intervention prevented the decreased acetylation of H3K9, H3K14, H3K18 and H3K27 in the brain of Ames dwarf mice, suggesting that early-life events have long-term consequences on aging-related epigenetic changes, particularly in acetylation on histone H3.

### Histone H3 acetylation alterations in response to early-life GH treatment in white adipose tissues

Increased global acetylation [27] and decreased activation of histone deacetylases [28] during adipocyte differentiation indicates that acetylation plays an important role in the regulation of differential protein expression in adipose tissues. Further, H3K27ac, which is associated with ‘open’ chromatin and cis-regulatory activity, was enriched during adipocyte differentiation [29]. Plasma levels of adiponectin, which promotes adipocyte differentiation, was much higher in Ames dwarf mice than in WT controls [1]. Our current data show that acetylation of H3K18 is increased in visceral adipose tissue of Ames dwarf mice, suggesting that this may have been due to adiponectin-related effects and slower aging of dwarf mice. Aging is known to impair adipocyte differentiation and alter adipose tissue-specific acetylation [30]. Intriguingly, our data of H3 acetylation in visceral adipose tissue of long-lived Ames dwarf mice is the further evidence that Ames dwarf mice are biologically younger than chronologically age-matched control mice. Acetylation of H3K9 was reduced in subcutaneous adipose tissue in male Ames dwarf mice, suggesting that an interaction of histone H3 acetylation on various residues regulates aging-related adipocyte differentiation in animals, which age at a different rate [31]. Moreover, early-life GH intervention further activated acetylation on 9, 14, 18 and 27 residues of histone H3 in Ames dwarf mice of both sexes, suggesting that actions of GH at early age can enhance the capacity of adipocyte differentiation in adult life.

### Summary

Histone modifications, particularly in the lysine residues of histone H3, have been implicated in the action of genetic and pharmaceutical interventions which extend longevity and health span in multiple model organisms. In this study, we found suppression of H3K4me and increase of H3K27me in both liver and brain tissues of hypo-pituitary Ames dwarf mice. Importantly, this pattern was altered by early-life GH treatment which was previously shown to resume (normalize) many adult phenotypic characteristics of these animals. Histone H3 was acetylated on multiple lysine sites in a tissue-specific manner and activation of histone H3 acetylation was influenced by GH intervention at early age. Genome-wide coordinated histone modifications on acetylation and methylation play an important role in the regulation of gene expression during aging. Therefore, this study serves as an initial, but important step in elucidating the epigenetic mechanisms by which early-life hormonal intervention can influence aging and longevity in mammals.

## MATERIALS AND METHODS

### Antibody and chemicals

Antibodies (Abs) for H3K4me3 and H3K27me3 were purchased from Abcam (Cambridge, MA, USA); Abs for H3K9ac, H3K14ac, H3K18ac, H3K27ac, H3K56ac, Histone H3 and GAPDH from Cell Signaling Technology (Danvers, MA, USA); HRP-linked anti-rabbit and HRP-linked anti-mouse secondary Abs, western blot stripping buffer, protease inhibitor tablet, phosphatase inhibitor tablet and PowerUp SYBR green master mix for real-time quantitative PCR from Thermo Fisher Scientific (Waltham, MA, USA). RNeasy plus kit was ordered from Qiagen (Hilden, Germany). LunaScript RT SuperMix Kit from NEB (Ipswich, MA, USA).

### Animals and growth hormone intervention

As previously reported [1], 2 week-old male and female Ames dwarf mice were subjected to porcine GH intervention (3 μg/g bw/time, twice/day) or saline (as vehicle) via s.c. injection for 6 weeks, and heterozygous siblings (df/+) of Ames dwarf mice phenotypically indistinguishable from WT were used as controls. During whole feeding process (before and after injection), the mice were maintained on a 12-hour light-dark cycle with ad libitum access to food (Rodent NIH31 Open Formula Auto with 18% protein and 4 % fat, ZEIGLER, Gardners, PA, USA) and water. Mice were euthanized for tissue collection at 20 months old. Animal protocols were approved by the Animal Care and Use Committee of Southern Illinois University and the University of Alabama at Birmingham.

### RNA extraction and real-time quantitative PCR

Tissue-specific RNA was extracted using RNeasy plus kit. 1 mg of RNA was reverse transcribed with LunaScript RT SuperMix Kit. Real-time quantitative PCR was performed in Applied Biosystems QuantStudio 3. Expression of beta-actin and GAPDH was used to normalize gene of interest in each sample [32].

The following primers were used to amplify cDNA following reverse transcription.

Dnmt1: 5’-TTGAAACTTCACCTAGTTCCGTGGC-3’ (F) and 5’-CTGCAGCACCACTCTCTGTGTCTAC-3’ (R);

Dnmt3α: 5’-CGCGATTTCTTGAGTCTAACCCCGT-3’ (F) and 5’-CTATTCTGCCGTGCTCCAGACACTC-3’ (R);

Dnmt3β: 5’-CACACTCTGGAGAAAGCCAGGGTTC-3’ (F) and 5’-AGTCATTGGTTGTGCGTCTTCGACT-3’ (R);

EZH2: 5’-ACATCGTAAGTGCAGTTATTCCTTC-3’ (F) and 5’-TTTAGGTGGTGTCTTTATACGCTCA-3’ (R);

beta-actin: 5’-TCTTTGCAGCTCCTTCGTTGCC-3’ (F) and 5’-CTGACCCATTCCCACCATCACAC-3’(R);

GAPDH: 5’-CCTGGAGAAACCTGCCAAGTATGATG-3’ (F) and 5’-AAGAGTGGGAGTTGCTGTTGAAGTC-3’(R).

### Protein extraction and western blot

As previously reported [33], tissue-specific protein was lysed on ice in RIPA buffer. Centrifugation at 14,800 rpm at 4° C for 20 min. The supernatant was collected and added to Laemmli sample buffer. Samples were boiled at 100° C for 5 min for western blots.

### Quantification of H3 methylation and H3 acetylation expression

Western blots were quantified using GeneTools from SYNGENE according to the manufacturer’s instructions. Histone H3 modification changes were calculated by dividing H3K4me3, H3K27me3, H3K9ac, H3K14ac, H3K18ac, H3K27ac, H3K56ac by Histone H3. Histone H3 modification changes were represented using the relative fold change of expression determined for all groups versus WT control (defined as 1.0).

### Statistical analysis

Statistical analyses were performed using Prism software (GraphPad, La Jolla, CA, USA). All data were shown as means ± SEM. One-way ANOVA (Newman-Keuls test) was performed. *p* < 0.05 was considered significant, * *p* <0.05 and ** *p* <0.01.

## Supplementary Material

Supplementary Figures
