# Supplementary material for: The effects of early-life growth hormone intervention on tissue specific histone H3 modifications in long-lived Ames dwarf mice
Source: Aging (Albany NY). 2020 Dec 28;13(2):1633–48. doi: 10.18632/aging.202451 (PMC7880366; doi:10.18632/aging.202451)
Supplement: Supplementary Figures [file aging-13-202451-s001.pdf]

SUPPLEMENTARY FIGURES

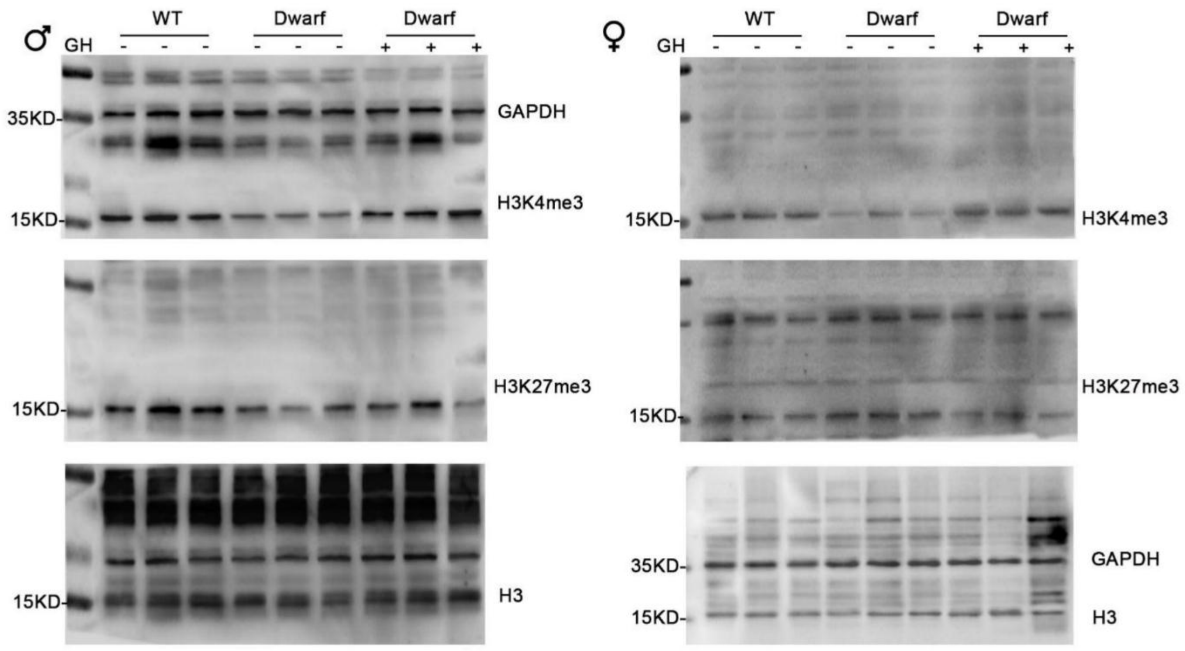

Supplementary Figure 1. Impact of early life GH intervention on hepatic expressions of H3 methylation on lysine 4 and 9 in Ames dwarf mice. Representative western blots in males (left) and females (right).

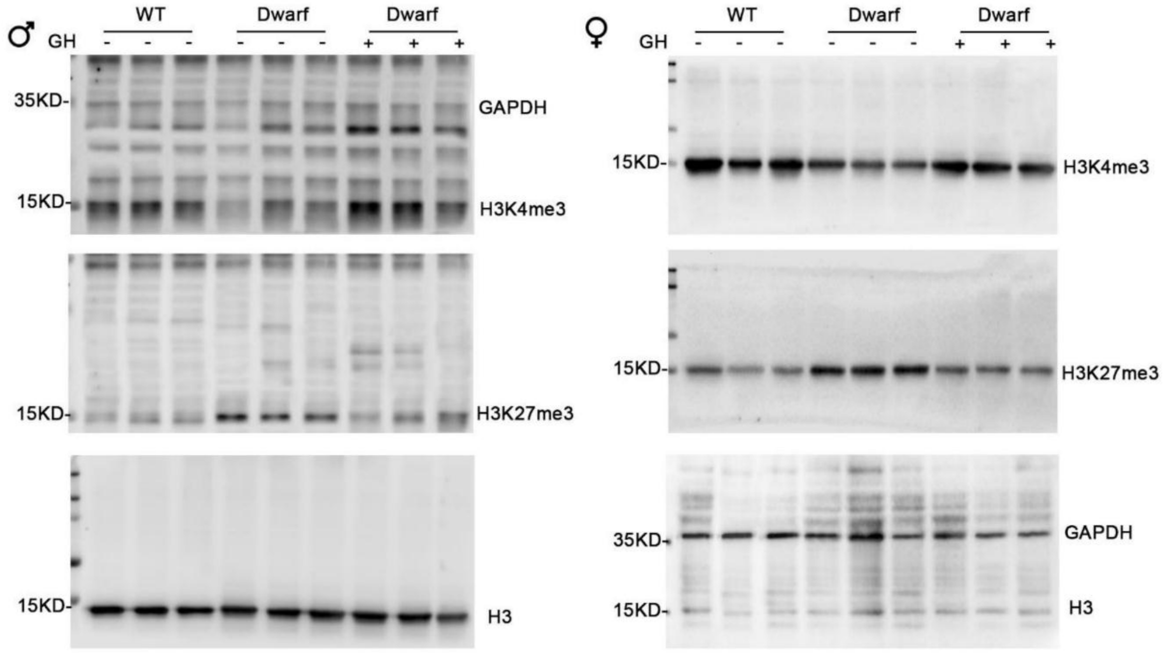

Supplementary Figure 2. Early-life GH intervention effects brain expression of H3 methylation on lysine 4 and 27 in Ames dwarf mice. Representative western blots in males (left) and females (right).

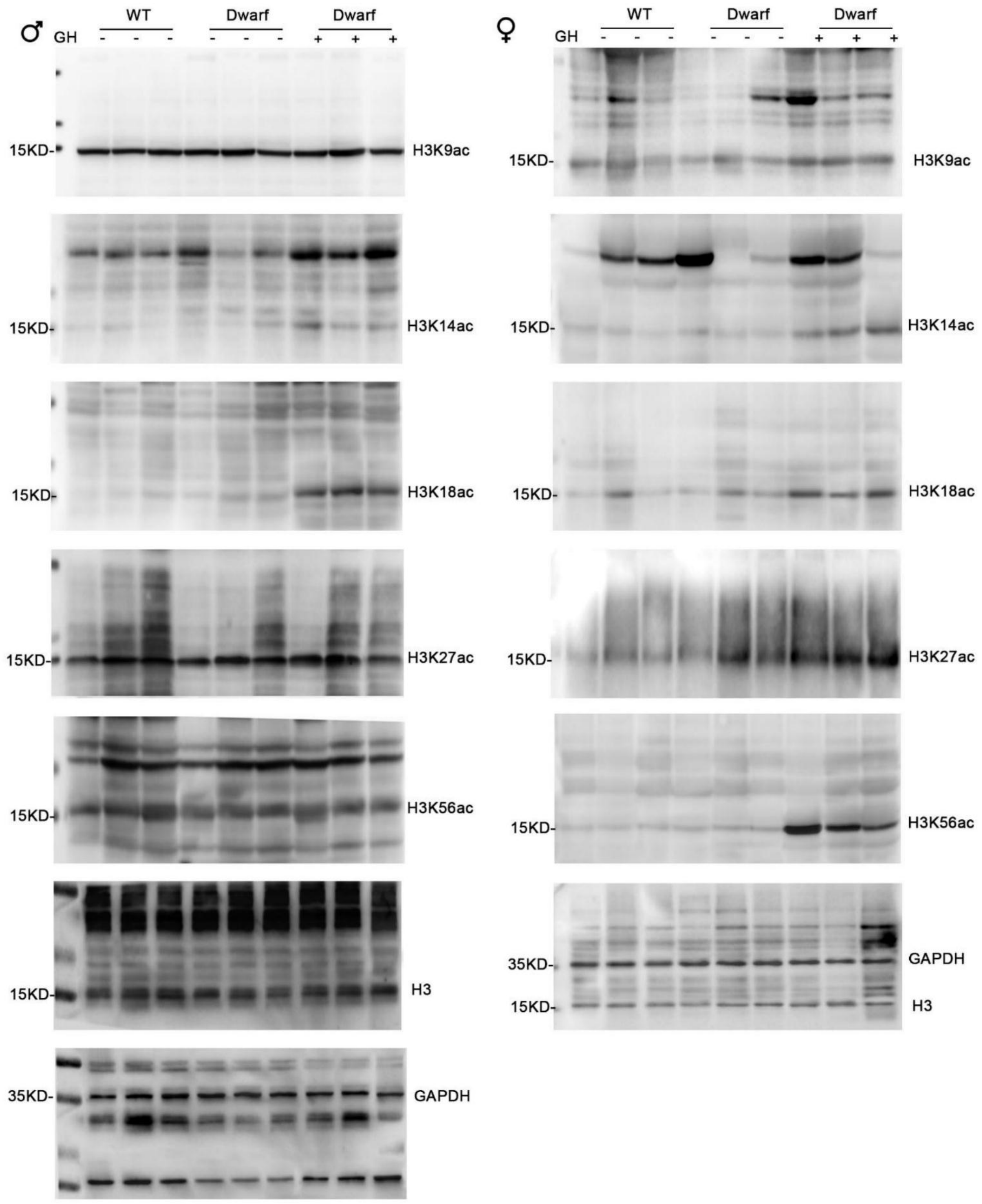

Supplementary Figure 3. Hepatic histone H3 acetylation changes in Ames dwarf mice upon early-life GH intervention. Representative western blots in males (left) and females (right).

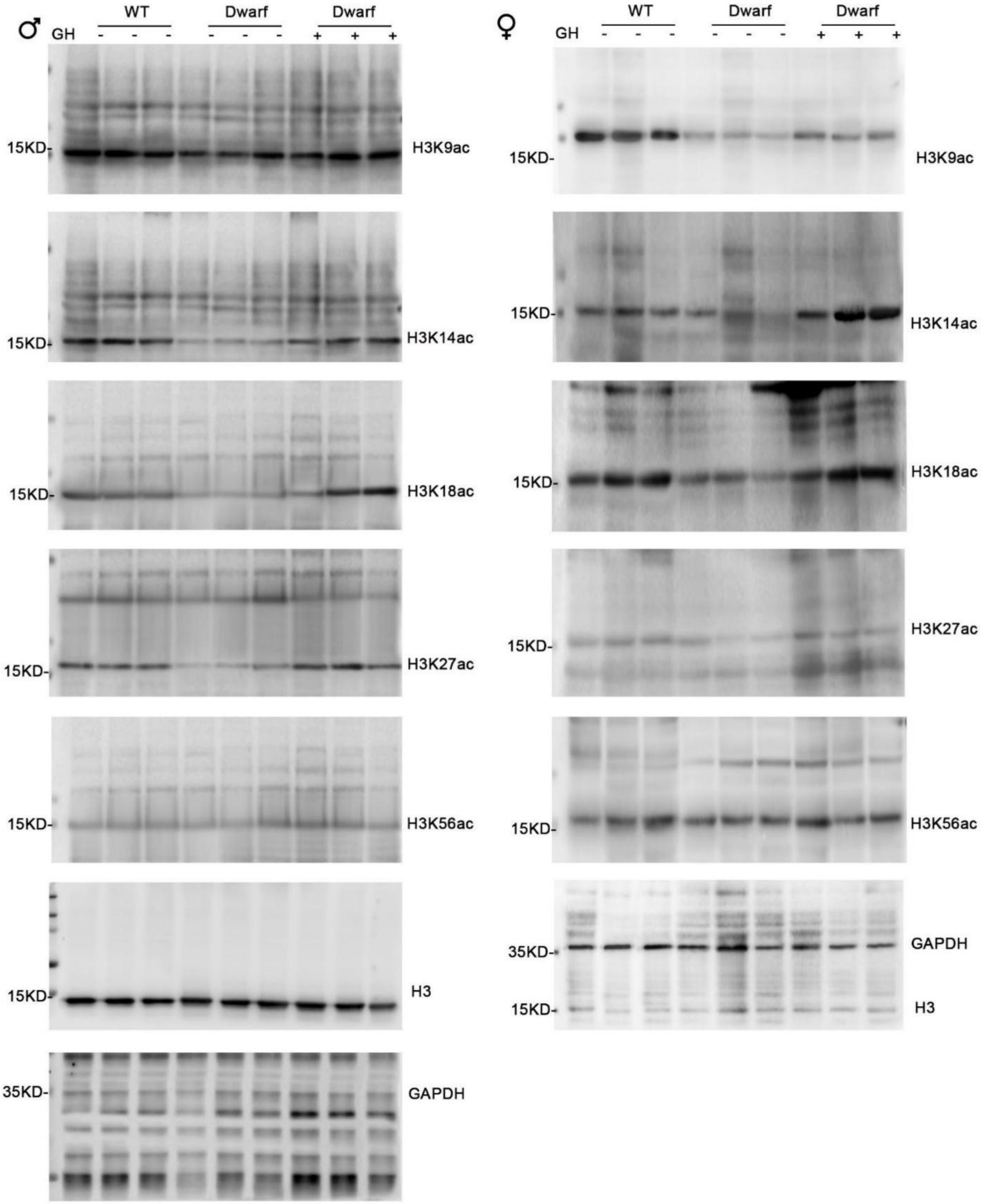

Supplementary Figure 4. Brain histone H3 acetylation altered in Ames dwarf mice upon early-life GH intervention. Representative western blots in males (left) and females (right).

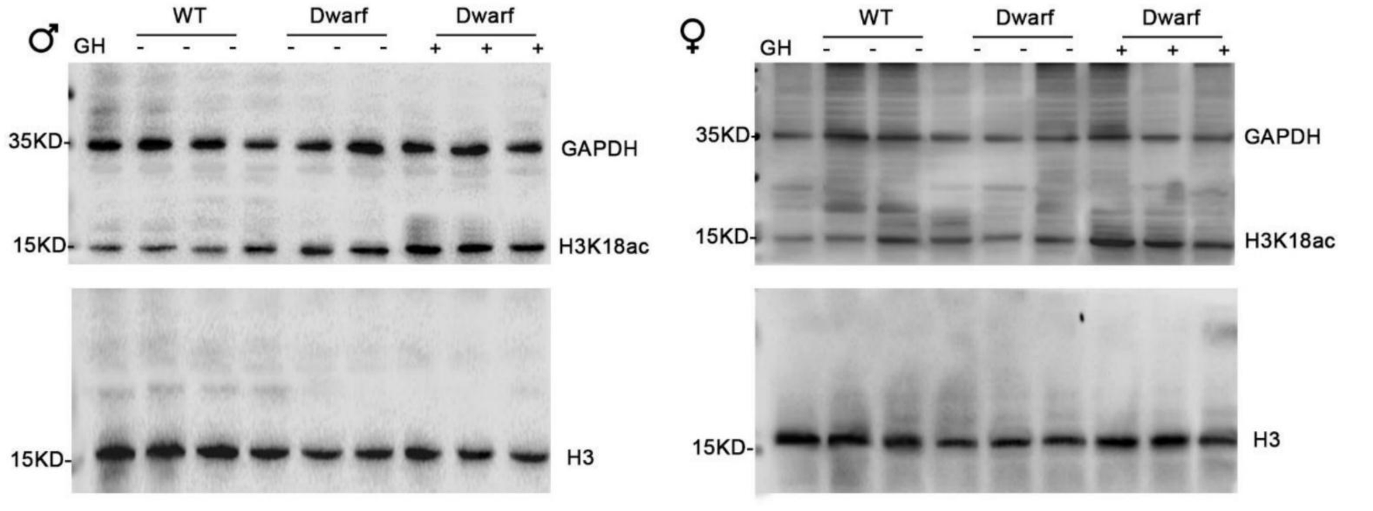

Supplementary Figure 5. Histone H3 acetylation changes in visceral adipose tissue of Ames dwarf mice upon GH intervention at early age. Representative western blots in males (left) and females (right).

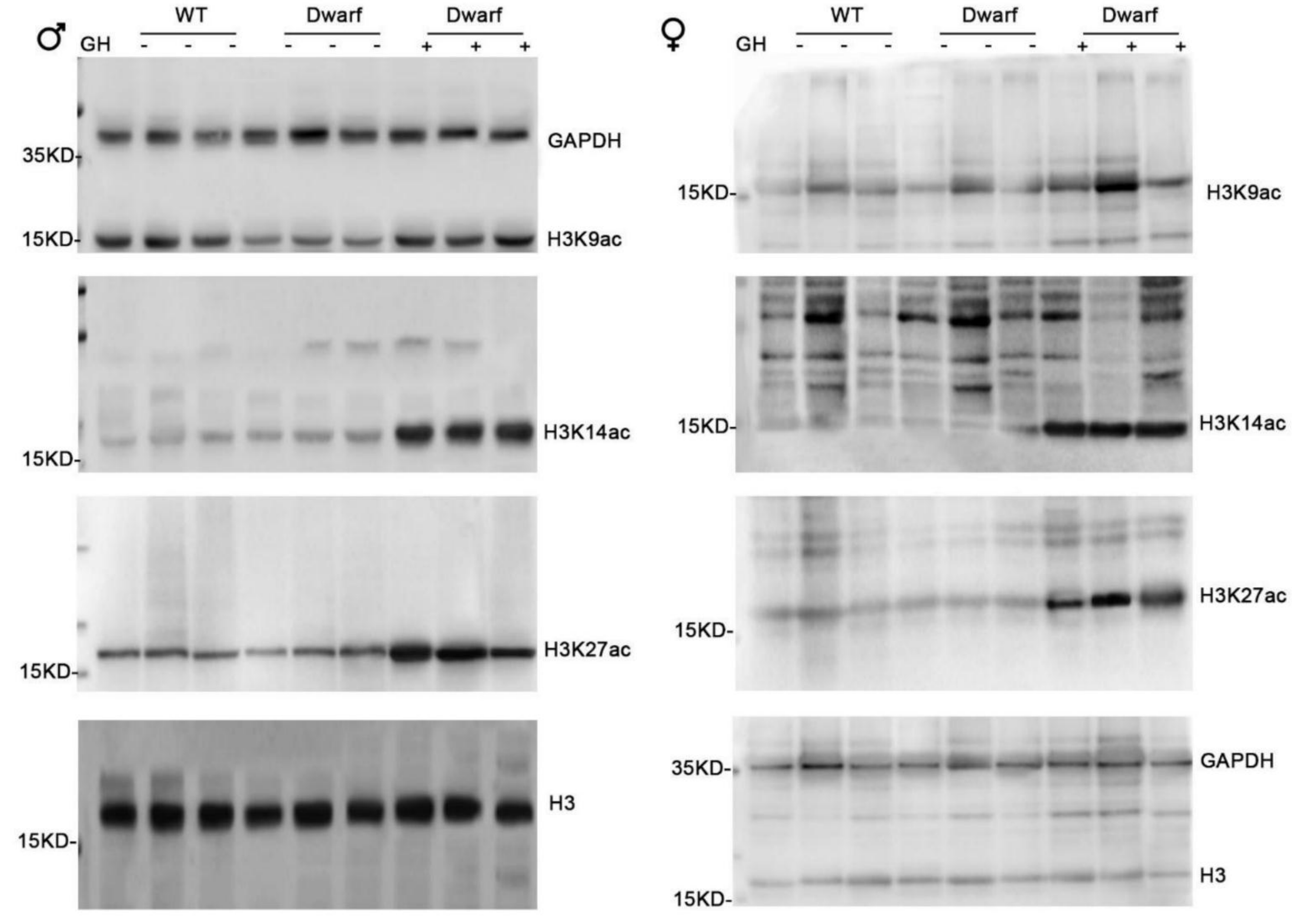

Supplementary Figure 6. Histone H3 acetylation changes in subcutaneous adipose tissue of Ames dwarf mice upon GH intervention at early age. Representative western blots in males (left) and females (right).
